# Role of Modulator of Inflammation Cyclooxygenase-2 in Gammaherpesvirus Mediated Tumorigenesis

**DOI:** 10.3389/fmicb.2017.00538

**Published:** 2017-03-28

**Authors:** Jaya Gandhi, Lohit Khera, Nivedita Gaur, Catherine Paul, Rajeev Kaul

**Affiliations:** Department of Microbiology, University of Delhi South CampusNew Delhi, India

**Keywords:** inflammation, gammaherpesvirus, COX-2

## Abstract

Chronic inflammation is recognized as a threat factor for cancer progression. Release of inflammatory molecules generates microenvironment which is highly favorable for development of tumor, cancer progression and metastasis. In cases of latent viral infections, generation of such a microenvironment is one of the major predisposing factors related to virus mediated tumorigenesis. Among various inflammatory mediators implicated in pathological process associated with cancer, the cyclooxygenase (COX) and its downstream effector molecules are of greater significance. Though the role of infectious agents in causing inflammation leading to transformation of cells has been more or less well established, however, the mechanism by which inflammation in itself modulates the events in life cycle of infectious agent is not very much clear. This is specifically important for gammaherpesviruses infections where viral life cycle is characterized by prolonged periods of latency when the virus remains hidden, immunologically undetectable and expresses only a very limited set of genes. Therefore, it is important to understand the mechanisms for role of inflammation in virus life cycle and tumorigenesis. This review is an attempt to summarize the latest findings highlighting the significance of COX-2 and its downstream signaling effectors role in life cycle events of gammaherpesviruses leading to progression of cancer.

## Introduction

Subsequent to primary infection, gammaherpesviruses follow two distinct life cycles in the human host: a latent infection in which the virus persists in a dormant state for long periods of host’s life, and a lytic form which results in release of infectious virions capable of *de novo* infection important for spread of virus to new hosts. The latent and lytic life cycles of gammaherpesviruses such as Kaposi’s sarcoma associated Herpesvirus (KSHV) and Epstein Barr Virus (EBV) are a result of a highly regulated interaction of the virus with its host. Understanding the regulation of switch between latency and lytic reactivation is an important problem in herpesvirus biology. Like other pathogenic viruses, EBV and KSHV- encoded genes have been shown to be involved in regulation of various cellular signaling cascades important for viral pathogenesis. One of the major cellular enzymes which are expressed during gammaherpesvirus directed malignancies is Cyclooxygenase-2 (COX-2). COX-2 is a key mediator of inflammatory pathways and its elevated expression has been found in several other human cancers as well. The relation between inflammation and cancer in general is well documented. Several recent studies on KSHV and EBV have pointed to the role of COX-2 in virus mediated tumorigenesis. This review is an attempt to summarize the latest findings highlighting the significance of COX-2 and its downstream signaling effectors role in life cycle events of gammaherpesviruses leading to progression of cancer.

## COX-2 Functions in Prostanoid Synthesis Pathway in which Downstream Effector PGE_2_ Act Via EP1-4 Receptors

**FIGURE 1:**
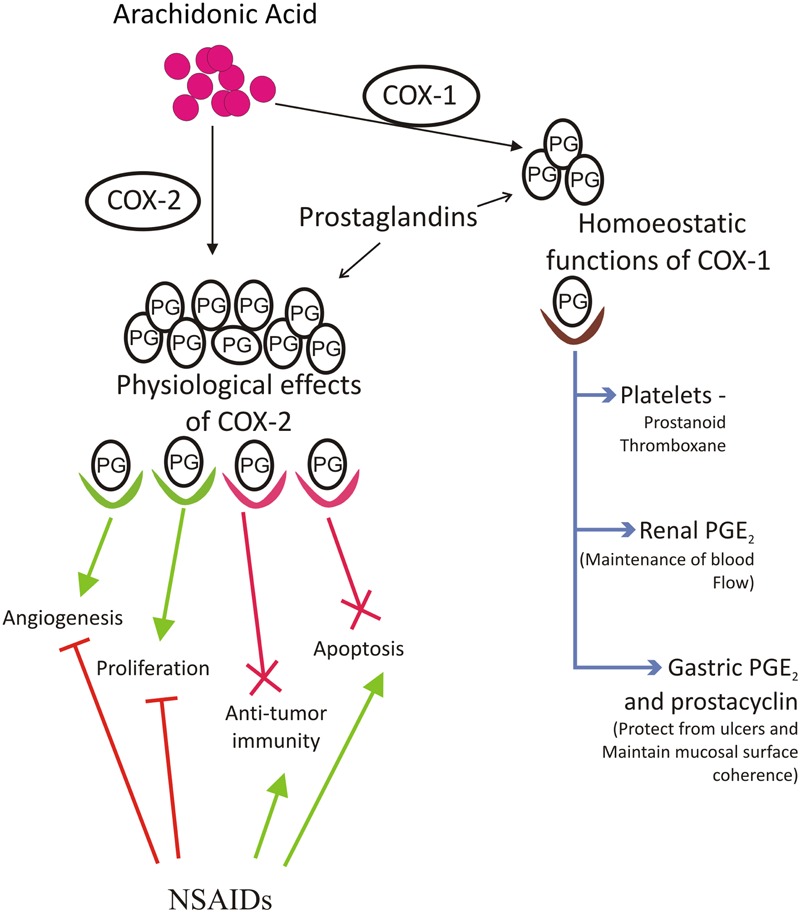
**Arachidonic acid is converted to prostaglandins by action of cyclooxygenase 1 and 2 Cox-1 and Cox-2 enzymes.** Cox-1 is important for maintaining homeostatic functions of body like platelet formation for blood, kidney development and its functions, maintenance of gastric mucosa etc. Cox-2 derived prostaglandin PGE_2_ is associated with increased inflammation, increased angiogenesis, greater metastatic and proliferative invasion, reduction in apoptosis and formation of immunosuppressive microenvironment. NSAIDs function as cox inhibitors and serve as effective tool against Cox mediated cancer. Aspirin and several other dual acting NSAIDs, which can block both Cox-1 and Cox-2 pathway, have several limitations and side effects associated with them and thus there was a need to develop Cox-2 specific inhibitors. NSAIDs reduce incidences of cancer by increasing apoptosis of tumor tissue, maintaining anti-tumor microenvironment, reducing proliferation and angiogenesis

Various inflammatory mediators implicated in pathological process associated with cancer include prostaglandins (PG), thromboxanes, and leukotrienes. Production of various prostaglandins is directed by coordinated activity of eicosanoid forming enzymes named Cyclooxygenase (COX). There are two isoforms of COX which are named as COX-1 and COX-2. COX-1 functions as a housekeeping isoform of cyclooxygenase and is constitutively expressed to serve functions such as control of renal blood flow, imparting protection to stomach against ulcers, production of prostaglandin E_2_ (PGE_2_) to maintain coherence and structure of gastric mucosal surface, and production of prostanoid thromboxane in platelets (Williams et al., 1999; Li et al., 2002; Leng et al., 2003) (**Figure 1**). COX-2 is an inducible early response gene and is activated in response to various extracellular or intracellular physiological stimuli. These include lipopolysaccharide (LPS), interleukin-1 (IL-1), tumour necrosis factor (TNF), epidermal growth factor (EGF), platelet activating factor (PAF), serum, endothelin, and arachidonic acid (Yucel-Lindberg et al., 1999; Medeiros et al., 2010; Font-Nieves et al., 2012). COX-2 over-expression metabolizes accumulation of PGE_2_. The downstream target molecules of PGE_2_ up-regulate several signaling pathways and down-regulate apoptotic proteins and hence contribute to various physiological processes including proliferation, survival, transformation, angiogenesis and metastasis (Satoh et al., 2012). The up-regulation and over-expression of COX-2 is mainly associated with inflammation, loss of apoptosis, uncontrolled cell proliferation, growth, metastasis, neovascularization, and angiogenesis finally leading to cancer. COX-2 generated prostaglandins have also been reported to function as immuno-suppressors. It has been shown that macrophage mediated and natural killer cell mediated cytotoxicity is suppressed by PGE_2_ (Williams et al., 1999; Leng et al., 2003). The precursor molecule for prostanoids is arachidonic acid, which is a 20 carbon unsaturated omega-6 fatty acid, usually esterified at SN-2 position of phospholipids and dispersed throughout the lipid bilayer of the cell membrane (Wang et al., 2007). In response to various stimuli such as growth factors, hormones, and cytokines; arachidonic acid is liberated from membrane and metabolized to various bioactive lipids. This conversion involves three major steps. The first step involves action of phospholipase A_2_ enzyme (secretory or cytoplasmic) on phospholipids resulting in the release of arachidonic acid. The second step involves addition of two molecules of oxygen to arachidonic acid forming bicyclic peroxide prostaglandin G_2_ (PGG_2_), an unstable intermediate. Lastly, PGG_2_ diffuses to the requisite site where peroxidation leads to reduction of unstable PGG_2_ to stable prostaglandin H2 (PGH_2_) which is converted to PGE_2_ by the enzyme PGE_2_ synthase (Smith, 1992; Park et al., 2006).

In different types of tumors, the COX-2 regulated downstream product PGE_2_ acts through prostaglandin E_2_ receptors (EP) named EP1, EP2, EP3 and EP4 (Konger et al., 2005). These receptors belong to family of G-protein coupled receptors (Narumiya et al., 1999; Tober et al., 2006). Despite close resemblance among all EP receptors, they exhibit different levels of binding affinity for PGE_2_ molecules. All four receptors are involved in activation of different intracellular signaling cascades. The role of individual EP receptors in tumorigenesis as well as malignancies has been extensively investigated. EP1 is closely associated with melanoma, carcinogenesis of colon and progression of breast cancer in late stage. It causes elevation in level of intracellular Ca^+2^ and induces its mobilization (Irie et al., 1994; Sugimoto and Narumiya, 2007). EP4 is coupled with phosphatidylinositol kinase and elevated cAMP levels (Hull et al., 2004). Antagonists of EP4 have been used in treatment of several disease of immune system (Yao et al., 2009), and as anti-inflammatory agents in inflammation associated diseases (Luschnig-Schratl et al., 2011).

## Inflammation is Closely Linked to Virus Mediated Cancers

Inflammation has been described as an immediate response of host’s immune system which plays a very important role in disease conditions. In general, viral infection evokes host’s immune system to eliminate the pathogen using inflammatory mechanisms. Inflammation, however, has also been recognized as one of the risk factors for progression of cancer (Sgambato and Cittadini, 2010). There is sufficient evidence to support the crucial role of inflammation in pathogenesis of several types of cancer including pancreatic, breast cancer, colorectal cancer, squamous cell carcinoma in head and neck, ovarian cancer, gastric adenocarcinoma, lung cancer, and hepatocellular carcinoma (HCC) (Wolff et al., 1998; Yip-Schneider et al., 2000; Cianchi et al., 2001; Costa et al., 2002; Fujiwaki et al., 2002; Gallo et al., 2002; Li et al., 2003; Tang et al., 2005). Several studies have shown that chronic infections may result in development of cancers (Ziegler and Buonaguro, 2009). Once the inflammatory pathways are activated, the cells release several pro-inflammatory factors including cytokines and chemokines to defend against the pathogen. It has been recognized that the release of inflammatory molecules can generate microenvironment which could be highly favorable for development of tumor, cancer progression and metastasis (Ouaguia et al., 2014). In cases of latent viral infections, generation of such a microenvironment is one of the major predisposing factors related to virus mediated tumorigenesis. Though the role of infectious agents in causing inflammation leading to transformation of cells has been more or less well established, however, the mechanism by which inflammation in itself modulates the events in life cycle of infectious agent is not very much clear. This is specifically important for gammaherpesviruses infections such as EBV and KSHV where viral life cycle is characterized by prolonged periods of latency when the virus remains hidden, immunologically undetectable and expresses only a very limited set of genes. The virus infection induced inflammatory response has been found to be associated with viral pathogenesis resulting in development of cancer. The entire process includes alterations in the cell, decreased cell death, propagation, invasion, neo-vascularization, and metastasis (Chen et al., 2013; Taniguchi et al., 2013). Therefore, it is important to understand the molecular mechanisms critical for role of inflammation in virus life cycle and tumorigenesis.

## EBV Infection is Often Associated with Elevated Expression of Modulator of Inflammation COX-2

The primary infection of EBV is followed by two distinct forms of life cycles in infected host; either a lytic infection resulting in release of progeny virions or the virus goes into long term latency in the infected cell. The transition between the latent and the lytic phases of the EBV life cycle operates through a highly efficient mechanism. The two phases of life cycle of EBV consists of interaction between EBV and its host which is tightly regulated, and may be separated into three important phases: (i) interaction of EBV with human B cells followed by uncontrolled multiplication of the infected cells; (ii) EBV entrance into the latent phase for persistent and lifelong infection; and (iii) EBV lytic reactivation from latent phase for the production of infectious virions capable of infecting similar cells or transmission of virus to a new host. Exposure to specific stimuli can also result in lytic reactivation of EBV in latently infected cells. Previous studies have shown that development and progression of EBV associated cancers such as nasopharyngeal carcinoma (NPC) and lymphoma is linked to lytic reactivation of EBV (Geser et al., 1982; Boos et al., 1987; Mueller et al., 1989; Lei et al., 2000; Lo, 2001; Liu et al., 2012). Extensive studies have been done to understand the biological importance of the up-regulated COX-2 level and subsequent increased level of PGE_2_ molecule and over-expression of EP receptors in development of malignancies associated EBV and KSHV (Paul et al., 2013c). The role of gammaherpesvirus infection in modulation COX-2, and the role of elevated COX-2 levels on the life cycle events of gammaherpesviruses are discussed ahead.

Several viruses induce COX-2 and PGE_2_ expression to enhance and establish efficient infection, although the details regarding cellular mechanisms explaining these observations remain mostly unexplored (Shelby et al., 2005). COX-2 has been constantly associated with gammaherpesviruses related malignancies. Increased COX-2 expression is a feature reported to be common to cancers associated with both EBV and KSHV infections (Shelby et al., 2005). COX-2 mediated inflammation is implicated in EBV induced tumorigenesis. Highly elevated COX-2 expression is a characteristic feature of EBV infected lymphoblastoid cell lines (LCLs) and EBV positive nasopharyngeal tumors when compared to EBV negative Burkitt’s lymphoma cells and EBV negative nasopharyngeal cancer. During pathological conditions, such as EBV associated Hodgkin lymphoma, over-expression of COX-2 is a critical factor (Al-Salam et al., 2013). A positive correlation has also been reported between EBV viral protein LMP1 and COX-2. LMP1 could mediate up-regulation of COX-2 hence accelerating lymph node metastasis in NPC (Bai and Tang, 2009). COX-2 has been found to be frequently expressed in tissue specimens of NPC which are LMP1-positive, whereas it was rarely detected in LMP1-negative NPC tissue (Murono et al., 2001; Fendri et al., 2008). The same study further showed that VEGF production in LMP-1 expressing cells was mediated by COX-2, suggesting COX-2 induction by LMP1 may play a role in angiogenesis in NPC. The LMP1 protein has been subsequently shown to upregulate COX-2 expression contributing toward cancer spread in lymph and progression of NPC (Yi et al., 2010). COX-2 is considered as a potential biomarker of EBV associated human malignant cancer (Diduk et al., 2012). Although increased amount of antibody titre against EBV have been reported in children suffering from asthma, a condition in which COX-2 levels within lung are elevated, no direct associations were seen between viral infections and the presence of allergen-specific IgE or asthma (Veiga et al., 2011). EBV infection was not found to be associated with COX-2 expression or survival in gastric carcinoma (Park et al., 2009). Direct link has also been reported between EBV latent protein EBNA3C and cellular metastatic repressor Nm23-H1 in modulating the expression of COX-2 enzyme (Kaul et al., 2006). In EBV positive cells the expression of COX-2 is much elevated as compared to EBV negative cells (Aggarwal et al., 2006; Kaul et al., 2006). Studies have shown that EBV infection activates STAT3 and NF-kappaB signaling pathways in NPC and upregulates pro-inflammatory cytokines and COX-2 expression, thus protecting infected cells from immune response and promote carcinogenesis (Lo et al., 2006). During lytic reactivation EBV transactivator protein Zta helps to evade immune surveillance. EBV Zta has been shown to enhance the activity of COX-2 promoter thereby upregulating the production of COX-2 and its downstream effector molecule PGE_2_ (Lee et al., 2011). It has also been shown that EBV could suppress PGE_2_ biosynthesis in LPS-activated monocytes (Savard et al., 2000). The data showed that inhibition of PGE_2_ by EBV was due to transcriptional downregulation of COX-2. It was further shown that the reduction in protein levels of COX-2 coincided with reduction in COX-2 mRNA transcript levels. Both LPS treatment and EBV infection did not affect COX-1 levels, indicating COX-2 as the major isoform involved in inflammatory stimuli induced PG synthesis in presence of EBV infection. However, a separate study showed that conditioned medium of Zta-expressing NPC cells enhances IL-10 production from monocytes which was mediated in part by elevated COX-2 levels in NPC cells (Lee et al., 2011). It has been speculated that the IL-10 production in monocytes may play role in facilitating local microenvironment in favor of immunosuppression (Lee et al., 2011).

## KSHV Infection is also Accompanied with Overexpression of COX-2

Kaposi’s sarcoma associated Herpesvirus positive tumor including multicentric castleman’s disease (MCD), primary effusion lymphoma (PEL), and Kaposi’s sarcoma (KS) exhibit higher expression of COX-2 (Shelby et al., 2005). Studies have shown that KSHV can induce robust COX-2 expression within hours in HMVEC-d cells and HFF cells, which increased for up to 72 h post infection (Sharma-Walia et al., 2006). KSHV infected cells also secreted PGE_2_ at higher levels in the culture supernatant medium. The study further showed that COX-2 was also induced by UV-irradiated KSHV but only at moderate levels indicating that KSHV gene expression was essential for elevated COX-2 expression. A study investigating the expression of COX-1 and COX-2 in classic and epidemic forms of Kaposi Sarcoma (KS) tissue showed that COX-1 and COX-2 were overexpressed significantly in classic and epidemic KS compared with control skin tissues suggesting that COXs may be involved in the pathogenesis of KS (Rossiello et al., 2007). The over-expression of COX-2 is also a constant factor in KSHV positive BC-3 cell lines (Paul et al., 2011). These findings suggest critical role for COX-2 mediated inflammatory pathway in EBV and KSHV mediated pathogenesis (Paul et al., 2013c). Studies on MHV-68 have shown that virus infection results in the induction of COX-2 protein and activation of the COX-2 promoter indicating association of MHV-68 with elevated COX-2 levels (Symensma et al., 2003).

## COX-2 and its Downstream Effectors Exert Critical Role in Tumorigenesis Progression Via Signaling Pathways that are also Regulated by Gammaherpesviruses

Several studies have shown a positive correlation between COX-2 or PGE_2_ expression and progression of different cancers including the cancers of lung, stomach and colon (Nadda et al., 2012; Shin et al., 2012). Studies have also shown that the depth of invasion and carcinoma development correlates with COX-2 over-expression in certain kinds of cancers (Coussens and Werb, 2002; Fujita et al., 2002). Elevated mRNA level of COX-2 has been detected in various human cancers like breast cancer, colorectal cancer, and prostate cancer (Xia and Kirkman, 1990; Chell et al., 2006; Biedrzycka et al., 2013). Over expression of COX-2 causes accumulation of downstream effector PGE_2_ which acts as a key molecule in maintaining tumor survival. It potentially increases tumor aggressiveness and inhibits apoptosis by various mechanisms. Many of the cellular pathways regulated by COX-2 are also regulated by EBV or KSHV coded proteins. Although it is not always clear how these interactions proceed, however, whatever is known may help in gaining new insights into role of chronic inflammation in gammaherpesvirus life cycle events leading to tumorigenesis.

Most evident effect of COX-2 downstream effector PGE_2_ which is seen on tumor cells is mediated by synthesis of metastasis promoting matrix metallo-proteinases (MMPs). MMPs are zinc-dependent proteolytic enzymes which are linked to different aspects of tumor progression, including cell migration, metastasis, and angiogenesis (Kessenbrock et al., 2010). EBV latent antigen EBNA3C has been shown to upregulate MMP-9 through interactions with the AP1 and NFkappaB transcription factors (Kuppers et al., 2005). Another EBV protein Zta has also been shown to upregulate MMP3 and MMP9 to promote cell migration and invasion (Lan et al., 2013). KSHV mediated notch1 activation also leads to upregulation of MT1-MMP promoting cancer cell metastasis (Cheng et al., 2011). An earlier study had shown that KSHV-infection of human umbilical vein endothelial cells (HUVEC) resulted in upregulation of MMP1, MMP2, and MMP9 promoting cell invasiveness (Qian et al., 2007). It has also been documented that over-expression of COX-2/PGE_2_ transforms replication in hepatitis B virus, cytomegalovirus as well as gammaherpesviruses (Tsujii et al., 1998; Nie and Honn, 2002; Zhu et al., 2002; Symensma et al., 2003). Studies have shown that COX-2 is stimulated in cancer which accelerates and intensifies tumor growth, tumor vascularization, angiogenesis, invasion and metastasis (Cianchi et al., 2001; Gallo et al., 2002; Li et al., 2003; Cheng et al., 2004). It is probable that COX-2 favors phenotypic changes that reduce apoptosis, thereby favoring tumor progression. Cells expressing increased COX-2 levels elucidated increased adhesion properties to extracellular matrix proteins (ECM) and also mediate resistance to apoptosis. EBV latent protein LMP1 is also known to mediate adhesion and motility to ECM in epithelial cells via integrin-a5 and N-cadherin (Wasil and Shair, 2015). These suggest that though it is possible that EBV mediated functions may have a role for COX-2; however, no study has shown a dire any direct evidence for this mechanism. There have been some studies which have investigated changes in protein profiles of ECM during the development of KS. Studies performed with human dermal microvascular endothelial cells (DMVEC) have shown that KSHV infection reduces expression of tropoelastin and fibulin-2 which are important ECM proteins (Alcendor et al., 2011; Alcendor, 2015).

It has been shown that COX-2 mediates increased expression of Bcl-2, though direct interaction between COX-2 and anti-apoptotic Bcl-2 protein has not yet been established. Interestingly, COX-2 inhibitors have shown to down regulate Bcl-2 protein expression suppressing tumorigenesis (Tsujii and DuBois, 1995). The interactions of EBV coded proteins with Bcl-2 protein family has been summarized elsewhere which show that most of the Bcl-2 family members are targeted by EBV-coded proteins (Fu et al., 2013). KSHV encodes a viral homolog of Bcl-2 named as KS-Bcl-2, which inhibits apoptosis and autophagy when over expressed in cancer cells have recently been found to be essential for virus replication (Gallo et al., 2017). The correlation between COX-2 and serine threonine kinase Akt signaling cascade has also been investigated and is believed to have significant implication in angiogenesis by promoting Akt activation (Gately, 2000). Infection of EBV or expression of EBV coded latent antigens can also result in activation of Akt1 via sphingosine kinase 1 (SPHK1) promoting cell migration (Lee et al., 2017). KSHV encoded microRNA miR-K12-3 (miR-K3) downregulation of G protein-coupled receptor kinase 2 GRK2 relieve its inhibition of AktT thereby activating Akt signaling (Hu et al., 2015). The microRNA miR-K3 has been recently shown to induce angiogenesis and promote viral latency (Li et al., 2016).

COX-2 has been also shown to generate immunosuppressive tumor microenvironment. Cooperative interaction between pro-inflammatory eicosanoids, cytokines, chemokines and carcinoma cells contribute to formation of immunosuppressive tumor microenvironment. PGE_2_ functions as immune modulator and plays a crucial role in maintaining microenvironment which favors tumor cell growth and invasion. It has been reported that PGE_2_ switches anti-tumor TH_1_ microenvironment to TH_2_ immunosuppressive microenvironment. It induces down-regulation of TH_1_ cytokines like TNFα, IFNγ, IL-2, IL-12, and upregulates TH_2_ cytokines such as IL-4, IL-10 which have immunosuppressive effect (Snijdewint et al., 1993; Kambayashi et al., 1995; Huang et al., 1998; Stolina et al., 2000). Studies on classical Hodgkins lymphoma (cHL) tissues investigating the percentage of infiltrating Tregs suggested that immune response suppression was important in both EBV positive and EBV negative cHL (Wu et al., 2016). KSHV vFLIP expression in mouse endothelial cells could influence myeloid differentiation leading to pro-inflammatory, angiogenic and immunosuppressive microenvironment (Ballon et al., 2015).

COX-2 downstream effector PGE_2_ has been shown to directly inhibit cytotoxic T cell activity. PGE_2_ up-regulates CD94/NKG2A heterodimer complex which is a natural killer receptor. Cross linking reaction between CD94 and T cells expressing this heterodimer prevents cytotoxic T cell activity (Zeddou et al., 2005). In a separate study, it has been reported that PGE_2_ indirectly eliminates anti-tumor effects of cytotoxic T cells. It inhibits dendritic cell maturation, down-regulates antigen presenting cells and causes abortive activation of naive CD8 (+) T cells (Ahmadi et al., 2008). CTL have been previously tested in clinical trials for prevention and treatment of EBV-associated lymphomas with promising results (Liu et al., 2002; Gallot et al., 2014). COX-2/ PGE_2_ mediated inhibition of CTL function can therefore result in promotion of tumorigenesis in EBV infected people with elevated COX-2 levels.

## COX-2 is Important During *De novo* Infection and Maintenance of Latency of Gammaherpesviruses

Murine herpesvirus 68 (MHV-68) can establish productive infections in many cell culture systems and help in better understanding of gammaherpesvirus replication and *de novo* infection. It has been used to investigate cellular responses to *de novo* viral infection and how they regulate gammaherpesvirus activity. A study which investigated COX-2 induction during MHV-68 infection suggested that COX-2 and PGE_2_ may have significant roles to play during *de novo* infection (Symensma et al., 2003). The study found that viral gene expression subsequent to MHV-68 infection induces COX-2 protein expression (Symensma et al., 2003). Their data also revealed that viral genes that were most upregulated by exogenous PGE_2_ were same which showed the biggest suppression following COX-2 inhibition by NS-398 treatment. It was observed that MVH-68 *de novo* infection induced COX-2 expression which mediated production of PGE_2_ which supported MHV-68 gene expression, indicating a clear role for COX-2 mediated pathway in MHV-68 pathogenesis. Earlier studies had indicated that COX-2 upregulation by KSHV is also important for latent gene expression (Sharma-Walia et al., 2006). The upregulation of COX-2 in early stage of KSHV infection and the induction of a moderate level of COX-2 by UV-irradiated KSHV and envelope glycoproteins suggested that COX-2 expression is initiated by initial attachment and entry steps of KSHV infection (Sharma-Walia et al., 2006). It has also been shown that KSHV gene expression early during infection and subsequent modulation of host genes are probably essential for the increased induction of COX-2 levels. The role of COX-2 in *de novo* infection of KSHV has subsequently been extensively investigated (Sharma-Walia et al., 2010b). That study showed that *de novo* KSHV infection induced COX-2 and m-PGES-1 in endothelial cells. The inhibition of COX-2 using NS-398 was found to reduce KSHV latent ORF73 gene expression in TIVE-LTC cells. In addition, the silencing of COX-2 reduced KSHV latent ORF73 gene expression in HMVEC-d cells indicating the importance of COX-2 in KSHV infection of host cells. KSHV induced COX-2 was also found to regulate the expression of a number of KSHV induced cytokines, and had a role in capillary tube formation induced by KSHV infection in endothelial cells. KSHV induced COX-2 was also found to regulate the activity of MMPs in *de novo* infected as well as in endothelial cells latently infected with KSHV. COX-2 induction subsequent to *de novo* KSHV infection could function through both autocrine and paracrine mechanisms and support HUVECs endothelial cell invasion. The study demonstrated that both PGE_2_ and COX-2 not only regulated inflammation associated processes by modulating secretion of cytokine but also controlled KSHV latency which is essential for viral genome maintenance and survival of host cell (Sharma-Walia et al., 2010b). Several transcription factors including Sp1, HIF-1α and AP-1 that are activated by PGE_2_ have been shown to modulate KSHV latency (ORF73) and lytic (ORF50) promoters. Therefore, it is possible for COX-2 and PGE_2_ to mediate effect on latency of KSHV through one or more among these transcriptional factors (Sharma-Walia et al., 2010b). Up-regulated COX-2 levels have been shown to be sustained by KSHV gene expression (Sharma-Walia et al., 2010a). The signaling molecules which are critical for KSHV entry can regulate promoter activity and transcription of COX-2, its protein expression and release of its downstream effector PGE_2_. The same study also showed that attachment and entry of KSHV into the target cells induces cross talk between different signaling pathways leading to transcriptional activation of COX-2 gene through its 5′ UTR region. This activates transcription factors like NFAT and CREB bound to the COX-2 promoter (Sharma-Walia et al., 2010a). One of the KSHV latent oncoprotein v-FLIP also induces host COX-2 protein and its downstream inflammatory metabolite PGE_2_ via NF-κB-dependent pathway to promote its tumorigenic effects indicating that COX-2 inhibitors may be exploited to block KSHV’s v-FLIP/K13 linked tumorigenesis (Sharma-Walia et al., 2012).

## COX-2 has Role in Latency-Lytic Switch of Gammaherpesviruses

**FIGURE 2:**
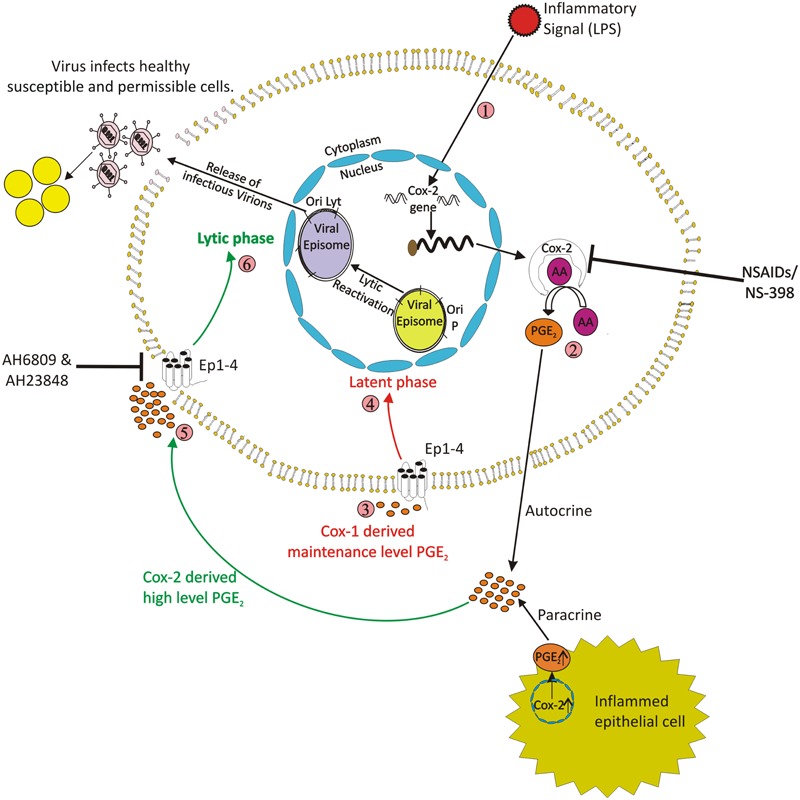
**Schematic model shows that COX-2 upregulation in response to an inflammatory signal can result in gammaherpesvirus lytic reactivation in latently infected cells.** The upregulation of COX-2 is associated with gammaherpesvirus lytic cycle reactivation. Inhibition of COX-2 with specific inhibitor NS-398 blocks lytic reactivation. The up regulation of COX-2 results in increased secretion of the downstream effector PGE_2_ which works both via autocrine and paracrine mode which is facilitated through EP receptors. The EP1 and EP4 receptors are also upregulated and their inhibition reduces viral lytic reactivation. Also, PGE_2_ released from inflamed distant epithelial cell also can act via a paracrine mode of action and lead to virus lytic reactivation in co-cultivated latently infected cell.

It has been earlier proposed that in gammaherpesvirus associated cancers, the infectious virions is required to be present for tumorigenesis (Sturzl et al., 2001). Many viruses, such as herpes simplex virus (HSV), human cytomegalovirus (HCMV), pseudorabies virus (PRV), human herpesvirus-6 (HHV-6), EBV, murine herpesvirus 68 (MHV-68), and human T-cell leukemia virus type 1 (HTLV-1), have been shown to induce COX-2 and release PGE_2_ that participate in viral lytic replication. It has been recently shown that COX-2 enzyme has a critical role in transition of EBV from latency to lytic reactivation in latently infected cells (Gandhi et al., 2015) (**Figure 2**). LPS treatment of EBV infected cells has been used to investigate role of upregulated COX-2 levels in latency-lytic switch of EBV (Gandhi et al., 2015). The study showed that the LPS treatment of gammaherpesvirus latently infected cells resulted in up regulation of COX-2 and also its downstream effector PGE_2_. The increase in levels of COX-2 and PGE_2_ levels was simultaneous to expression of EBV gp350 protein which is a key late lytic protein expressed during virus assembly. This was accompanied with detection of EBV virion in cell culture supernatant and increased cell death indicating occurrence of lytic reactivation in a significant fraction of EBV infected cells. The Akata cells, which are EBV latently infected Burkitt’s lymphoma cells also showed similar results suggesting a strong possibility of this phenomenon to have biological relevance *in vivo*. Thus, it could be inferred that COX-2 plays a direct and important role in the viral lytic reactivation. Moreover, the lytic reactivation mediated by over-expression of COX-2 generated intact and biologically infectious progeny virions which could infect and transform PBMCs. That study also showed the importance of PGE_2_ in EBV lytic reactivation. PGE_2_ works in autocrine and paracrine mode and thus is highly important during episodes of chronic inflammation leading to development and progression of cancer. There is a high possibility that chronic inflammation in one part of body may lead to EBV lytic reactivation of latently infected cells sitting at another part of the body due to paracrine activity of prostaglandins generated due to inflammation. The progeny virions thus released may infect naïve cells thereby increasing the probability of transformation and tumorigenesis. In immune-compromised individuals, such phenomenon may lead to increased risk of generation of gammaherpesvirus related malignancies in people with chronic inflammatory conditions with elevated COX-2 levels.

## COX-2 Downstream Effector PGE_2_ and EP Receptors Role in Latency-Lytic Switch

The PGE_2_ molecule binds to its specific receptors EP1 and EP4, which are upregulated during the episodes of several human malignancies. The overexpression of COX-2 and PGE_2_ in LPS treated EBV infected cells has been shown to be simultaneous to overexpression of PGE_2_ receptors EP1 and EP4 (Gandhi et al., 2015). The significance of biological functions mediated by COX-2 downstream signaling via PGE_2_ and EP receptors has also been well studied in different virus associated tumors specially in tumors related with oncogenic human herpes virus (Paul et al., 2013b). The PGE_2_ downstream EP receptors are G-protein coupled receptors which mediate and regulate biochemical changes involving the immune system (De Keijzer et al., 2013). The inhibition of EP1 and EP4 receptors using chemical inhibitors significantly reduces lytic reactivation of EBV even when COX-2 levels are up-regulated. It may be emphasized that lytic reactivation of virus in EBV or KSHV latently infected cells is commonly initiated by using a histone deacetylase inhibitor Sodium butyrate (NaB) and a histone acetyltransferase inducer TPA (Luka et al., 1979; Daibata et al., 1998; Miller et al., 2007; Zhang et al., 2014). Sodium butyrate and TPA treatment activates EP receptors linked intracellular signaling pathways (Shelby et al., 2005). TPA activates PKC via EP1 and Sodium butyrate activates the PKA via EP2 and EP4 pathway (Shelby et al., 2005). EP receptors regulate distinct intracellular signaling pathways that result in the increase in calcium levels inside the cell (Reader et al., 2011). The epigenetic regulation of EP receptors functions has also been previously reported (Gray et al., 2009). The intracellular PGE_2_ levels can also promote cancer cell apoptosis (Lalier et al., 2011). Therefore, it is entirely possible that similar pathways downstream of receptors EP1 and EP4 are exploited in COX-2/PGE_2_ mediated lytic reactivation of EBV and KSHV, which may need further studies.

Cellular protein Nuclear factor E2-related factor 2 (Nrf2) mediates global lytic gene repression via interaction with the host transcriptional repressor KAP1 and another viral protein, latency-associated nuclear antigen (LANA-1) in KSHV infected cells. Hence Nrf2 has an important role in viral gene expression of KSHV, its lytic reactivation, and survival of infected cell (Gjyshi et al., 2015). It has been recently shown that Nrf2 transcription and protein levels are induced during KSHV latency via COX-2/PGE_2_/EP4/PKC signaling. KSHV lytic reactivation can be induced both by Nrf2 knockdown as well as brusatol-mediated inhibition in latently infected cells. Another study has identified MAP4K4 which is a STE20 kinase family member, as a modulator of KSHV lytic cycle and invasive phenotype of KSHV-infected endothelial cells. MAP4K4 has been linked to COX-2, which also contributes to KSHV lytic replication. MAP4K4-dependent COX-2 expression and enzymatic activity is required for successful reactivation of KSHV and the invasiveness of KSHV-infected endothelial cells (Gjyshi et al., 2015).

## Clinical and Therapeutic Implications

The studies on co-cultivation experiments has demonstrated that lytic reactivation in EBV latently infected lymphoblastoid cells can be induced by co-cultured epithelial cells with up-regulated COX-2 levels (Gandhi et al., 2015). Mere adding of culture supernatant from epithelial cells over-expressing COX-2 or exogenous pure PGE_2_ was sufficient to induce EBV reactivation. The progeny virions released from cells upon lytic reactivation were also biologically active and functional when tested for their ability to infect and transform freshly isolated PBMCs. These observations may explain how COX-2 can possibly contribute to the incidences of EBV associated cancers in patients suffering from chronic inflammation. The upregulated COX-2 levels due to inflammation may induce PGE_2_ release which may act on EBV latently infected cells. The lytic reactivation of virus will release progeny virions which may then infect naïve cells *de novo*. If immune system is compromised, this may substantially increase probability of transformation and tumorigenesis, as is clinically apparent as well. Interestingly, it has also been previously suggested that triggering EBV lytic reactivation may be of use as a therapeutic intervention. EBV lytic genes’ expression in tumor cells may induce strong immune recognition, and hence help in killing tumor cells (Giunco et al., 2013). When the role of COX-2 in primary effusion lymphoma (PEL) using nimesulide, a COX-2 specific nonsteroidal anti-inflammatory drug (NSAID) was examined, it was found to be efficacious in inducing proliferation arrest in EBV positive as well as KSHV positive cells (Paul et al., 2011). The use of antagonists against EP1, EP2, and EP4 has been shown to downregulate proliferation of KSHV positive and EBV positive cells in culture (Paul et al., 2013a). Concurrent targeting of COX-2 and EP1/EP4 has been reported to have anti-cancer effects due to the simultaneous inhibition of viral and non-viral mediated tumorigenic mechanisms acting at multiple levels such as viral-host protein interactions, host and viral gene expression through regulation of epigenetic mechanisms such as methylation, host signaling, immune system activation, pro-inflammatory and cell survival processes (Paul et al., 2013a). In addition to COX-2 being a therapeutic target for KSHV associated malignancies, EP receptors may represent ideal targets for pharmacologic agents as PGE_2_ analogs and their blockers/antagonists possess antineoplastic activity, without the reported gastrointestinal and cardiovascular toxicity observed with few a NSAIDs (Paul et al., 2013c).

## Conclusion

Numerous studies have shown that the viral infection induced inflammatory response is associated with pathogenesis of the virus leading to transformation of infected cells, which is followed by increased survival, proliferation, invasion, and other pro-cancerous effects like angiogenesis and metastasis. The role of inflammation, if any, in regulating events in pathogenic virus’ life cycle is, however, not clearly understood. The importance of the inflammatory response of host needs careful evaluation in the case of gammaherpesviruses such as EBV and KSHV, which remain latent for long time periods and subsequently undergo reactivation. Virus reactivation is an important step in the infection cycle of gammaherpesviruses which is critical for dissemination of virus to novel hosts and *de novo* infection of nascent cells. The infection may result in transformation and tumorigenesis in the immuno-compromised hosts. The upregulation of COX-2 expression and its downstream effector molecules play a key role in life cycle events of EBV as well as KSHV. A link between up-regulated COX-2 levels and induction of lytic reactivation in gammaherpesvirus infected cells has been recently shown. It is known that patients with chronic inflammatory conditions with up-regulated COX-2 levels show high incidences of EBV associated malignancies indicating role of elevated COX-2 in virus mediated tumorigenesis. Recent studies and observations add new horizon towards deeper understanding of the relation of inflammation with the progression of oncogenic gammaherpesviruses mediated cancers.

## Author Contributions

All listed authors have made substantial, direct and intellectual contribution to the work, and have approved it for publication.

## Conflict of Interest Statement

The authors declare that the research was conducted in the absence of any commercial or financial relationships that could be construed as a potential conflict of interest.
